# Effect of the Addition of Dandelion (*Taraxacum officinale*) on the Protein Profile, Antiradical Activity, and Microbiological Status of Raw-Ripening Pork Sausage

**DOI:** 10.3390/molecules29102249

**Published:** 2024-05-10

**Authors:** Karolina Wójciak, Małgorzata Materska, Arkadiusz Pełka, Agata Michalska, Teresa Małecka-Massalska, Miroslava Kačániová, Natália Čmiková, Mirosław Słowiński

**Affiliations:** 1 Department of Animal Food Technology, Faculty of Food Science and Biotechnology, University of Life Sciences in Lublin, Skromna 8, 20-704 Lublin, Poland; arek281999@wp.pl (A.P.); agata.michalska@up.lublin.pl (A.M.); 2 Department of Chemistry, Faculty of Food Science and Biotechnology, University of Life Sciences in Lublin, Akademicka 15, 20-950 Lublin, Poland; malgorzata.materska@up.lublin.pl; 3 Department of Human Physiology, Medical University of Lublin, Radziwiłłowska 11, 20-080 Lublin, Poland; teresa.malecka-massalska@umlub.pl; 4 Institute of Horticulture, Faculty of Horticulture and Landscape Engineering, Slovak University of Agriculture, Tr. A. Hlinku 2, 94976 Nitra, Slovakia or miroslava.kacaniova@gmail.com (M.K.); n.cmikova@gmail.com (N.Č.); 5 School of Medical & Health Sciences, University of Economics and Human Sciences in Warsaw, Okopowa 59, 01-043 Warszawa, Poland; 6 Division of Meat Technology, Department of Food Technology and Food Evaluation, Institute of Food Sciences, Warsaw University of Life Sciences—SGGW, 02-787 Warsaw, Poland; miroslaw_slowinski@sggw.edu.pl

**Keywords:** natural antioxidant, *Taraxacum officinale*, LCMS-ESI-QTOF, microbiological quality, oxidation

## Abstract

The study evaluated the effect of adding dandelion extract on the characteristics of raw-ripening pork sausages while reducing the nitrite addition from 150 to 80 mg/kg. The sausages were made primarily from pork ham (80%) and pork jowl (20%). The process involved curing, preparing the meat stuffing, forming the links, and then subjecting the sausages to a 21-day ripening period. Physicochemical parameters such as pH, water activity, and oxidation-reduction potential were compared at the beginning of production and after the ripening process. The study also examined the impact of ripening on protein metabolism in pork sausages and compared the protein profiles of different sausage variants. The obtained research results indicate that dandelion-leaf extract (*Taraxacum officinale*) were rich in phenolic acids, flavonoids, coumarins, and their derivatives (LC-QTOF-MS method). Antiradical activity test against the ABTS^+*^ and DPPH radical, and the TBARS index, demonstrated that addition of dandelion (0.5–1%) significantly improved the oxidative stability of raw-ripening sausages with nitrite content reduction to 80 mg/kg. A microbiological evaluation of the sausages was also carried out to assess the correctness of the ripening process. The total number of viable bacteria, lactic acid bacteria, and coliforms were evaluated and subsequently identified by mass spectrometry.

## 1. Introduction

Today’s consumers demonstrate a clear preference for foods generally perceived as healthy. This is dictated, among others, by the spread of knowledge about the impact of food and certain dietary patterns on the occurrence of chronic diseases [1]. Much of the literature has focused on strategies to improve the nutritional value and quality of meat products. One of the directions in the development of meat science has been to reduce the impact of oxidation processes in meat to ensure product safety and quality throughout its shelf life. Meat, the fundamental raw material in the meat industry, is susceptible to deterioration due to chemical and biological reactions, in particular oxidative changes. These processes begin during technological processing when muscle cell membranes are disrupted, allowing prooxidants to attack the double bonds found in unsaturated fatty acids. This results in the production of lipid peroxyl radicals and other oxygen species through free radical chain reactions [2]. These lipid changes, initiated by free radical activity, contribute to the formation of secondary products such as aldehydes, ketones, and esters, which can negatively affect the sensory and nutritional properties of meat products [3]. Undesirable oxidative changes in muscle tissue can be inhibited by incorporating phytophenols (anthocyanins, flavonoids, and other phenolic compounds) in the processing [4,5,6]. It has been proven that phytophenols stabilize the color of meat and inhibit the formation of unpleasant aftertastes resulting from oxidation, improving the taste of meat products. The extensive literature on the use of so-called “natural antioxidants” covers a wide range of plant sources (whole plants and their components dried, plant extracts, and freeze-dried plants), various meat products, and concentrations and dosage methods for meat products. This is particularly important, as reports of pro-oxidant effects of plant phenols in meat systems have also been noted [7,8,9]. The antioxidant properties of dandelion are mainly attributed to its polyphenolic compounds. Ethanolic extracts of dandelion leaves contain approximately three times more phenolic compounds (9.9%) and flavonoids (0.086%) than extracts from the root [10,11], which are primarily responsible for its antioxidant activity. Other antioxidant compounds in dandelion include alkaloids, steroids, terpenoids, glycosides, reducing sugars, and tannins. The addition of 10% powdered dandelion leaf extract was found to affect the functional properties of chicken meat loaves in particular, improving their antioxidant potential [12]. Similarly, Choi et al. [13] suggested that dandelion leaf extracts could be beneficial in stabilizing the color, flavor, and antioxidant potential of ground pork. The effect of adding *Taraxacum officinale* on the antioxidant and angiotensin-converting enzyme inhibitory properties of protein extracts from sous-vide beef marinated in sour milk with simultaneous ultrasound application was also studied. The study demonstrated that adding dandelion to the meat product could further enhance the antioxidant effect of peptides [14].

Another direction for the application of plant raw materials in meat products is to replace certain additives that, while effective and safe in permitted doses, may raise consumer concerns. Nitrites and nitrates are involved in the formation of carcinogenic N-nitrosamines [15], necessitating their limited use as preservatives [16]. In previous studies, we demonstrated the feasibility of producing a meat product with reduced nitrite content while fortifying it with a plant additive black currant (*Ribes nigrum* L.) willow herb (*Epilobium angustifolium* L.) extract without compromising the quality and health safety of the product [17,18,19]. This study was aimed at investigating the potential for reducing oxidative changes in raw-ripening sausage by lowering nitrite addition from 150 to 80 mg/kg, without adversely affecting the oxidative stability and microbiological quality of the product.

It has been elucidated that phenolic compounds can interact with proteins, primarily through the amino groups of the side chain, either directly through noncovalent (reversible) interactions or through covalent bonds (irreversible, usually in alkaline or oxidizing environments), enhancing the crosslinking ability of proteins [20]. Phenolic compounds promote the formation of protein complexes by converting free sulfhydryl bonds to disulfide bonds. Quinones, on the other hand, act as bridges for protein dimerization or polymerization. These changes can be observed through electrophoresis, which separates proteins based on their size [21].

According to our hypothesis, the reduction of nitrite in ripening pork sausages can directly affect the oxidation processes of fats and proteins, and consequently, the antioxidant potential of biomolecules (e.g., peptides) in the meat product. The impact of white mulberry polyphenols on the stability of oxidation, including proteins, in dried minced pork slices, a popular Chinese meat product, was studied by Xu et al. [22]. The authors demonstrated that the addition of white mulberry significantly reduced the increase in TBARS values and carbonyl formation, while also reducing the loss of sulfhydryl residues in vacuum-sealed, dried minced pork slices during a 20-day storage period at room temperature. Apart from the biologically active compounds from the plant additive, the protein and peptide content can also directly affect the antioxidant potential of the ripening meat product.

In this study, we analyzed the changes in the protein profile of raw-ripening pork sausages fortified with dandelion using an electrophoretic method. We also evaluated the antioxidant potential of the products. In this regard, in vitro tests were carried out to evaluate the ability to neutralize ABTS^+*^ and DPPH radicals. To determine the factor most conducive to the antiradical activity of meat products, we analyzed the alcohol extract (mainly containing antioxidants of plant origin), water extract (containing sarcoplasmic proteins in addition to plant antioxidants), and peptides. Furthermore, we measured the level of secondary fat-oxidation products to reinforce the observed trends in antioxidant characteristics. We discussed the evaluation of physicochemical parameters typical of the product, including pH, oxidation-reduction potential (ORP), and water activity, to monitor the ripening process over 82 days. Microbiological analyses of the samples evaluated were carried out to check food safety.

## 2. Results and Discussion

### 2.1. Assessment of Antioxidant Properties of Water Extract from Dandelion Leaves

The yield of the extraction process was high, exceeding 30%. Chemical analysis of the prepared extract demonstrated its rich composition and significant antiradical activity (Table 1). The antiradical activity of the tested extract, determined in the DPPH radical system and expressed as an EC_50_ value, was 68.4 μg/mL, which was three times lower than the activity of butylated hydroxytoluene (BHT) under the same conditions, with a value of 20.4 μg/mL.

A qualitative analysis of the dandelion leaf extract was performed using the LC-QTOF-MS method. It confirmed the presence of seven phenolic acids (compounds number 1, 2, 11, 15, 16, 18, and 20), eight flavonoids and their derivatives (compounds number 8, 9, 10, 13, 14, 17, 19, and 21), four coumarins and their derivatives (compounds number 3, 4, 5, and 12), and other two compounds typical for *Taraxanum* genus (compounds number 6 and 7) (Table 2). The identification of individual compounds is described in the Supplemental Material.

### 2.2. Assessment of Physicochemical Parameters during Ripening

The physicochemical transformation of meat is a dynamic process that begins as early as slaughter and significantly influences the taste, aroma, and texture characteristics of meat and meat products. Ripening products are a category of products in which these changes occur throughout the production period, without disruption from heat treatment that could inactivate enzymatic proteins, thereby affecting proteolysis, lipolysis, and other processes that occur in parallel, often over many months. These changes can also be promoted by exogenous enzymes of microbial origin from starter cultures used in meat fermentation. This complexity makes it challenging to strictly control the technological parameters of meat-ripening products. One approach to address this challenge is to monitor the trends in physicochemical parameter changes, which are the basic distinguishing features of the quality of meat products. Table 3 demonstrates the changes in acidity (pH), ORP, and water activity (aw) observed in ripening sausages over 81 days.

The analysis of the pH values of the products immediately after production (day 1) demonstrated statistically significant differences between the research variants (*p* < 0.05). The sausage with the highest amount of dandelion extract (P_80_M1; pH 6.55) exhibited the highest pH value (*p* < 0.05), while there were no significant differences among the other variants at this time (day 1). After 21 days of production ripening, the variants without plant additives (P_80, pH 5.44; P_150, pH 5.43) had significantly higher pH values (*p* < 0.05) compared to the other samples. The lowest statistically significant pH value was recorded in the variant with 1% dandelion addition (P_80_M1) (21 days of ripening). This trend persisted until the 51st day of ripening, while in the next test period (81st day), no effect of the applied technological treatments on the pH value of the products was observed (the differences between the variants were not statistically significant, *p* > 0.05).

The observed significant (*p* < 0.05) reduction in the pH value of sausages at the beginning of the production process was most likely due to intensive fermentation, during which carbohydrate metabolism associated with the production of organic acids by lactic acid bacteria occurred. Similar results were reported by Zhao et al. [23], Xing Tang et al. [24], Xiao et al. [25], and Yu et al. [26] in studies on the production of raw-ripening sausages with various combinations of starter cultures and spices. As reported in the literature, lowering the pH value below 5.3 is indicative of a properly conducted fermentation and production ripening process [27]. The pH value in all test variants stabilized by the 51st day of ripening, except for the P_80_M1 sample, which showed a statistically significant (*p* < 0.05) increase in the parameter. On the 81st day of ripening, the pH value increased significantly (*p* < 0.05) in all test variants, except for the control variant (P_150), where it remained constant. The significant increase in pH value was likely related to the release of peptides, amino acids, and ammonia due to proteolytic reactions activated by the meat enzymes and enzymes of bacterial origin. Compounds formed during the proteolytic decomposition of proteins demonstrated a buffering effect against organic acids produced by lactic acid bacteria during the fermentation process and refrigerated storage. This is supported by the findings of other authors [25,26,28,29,30]. In addition, the pH values observed in this study throughout the production period may enhance the high antioxidant potential of the product. Flores et al. [31] noted that the formation of peptides and amino acids is more intense at lower pH values due to the increased activity of selected enzymes. Therefore, dried meat products serve as a good source of large amounts of protein-derived components, including bioactive molecules with antioxidant activity.

The ORP potential values reflect the natural ability of a biochemical system to accept or donate electrons. A lower redox potential indicates a greater ability to donate electrons, thereby eliminating free radicals. ORP can thus indicate the presence of components with antioxidant properties that naturally protect against oxidative changes. Statistical analysis of ORP values in ripening pork sausages on the first day after production showed significant differences between the variants, confirming the influence of the technological treatment on the parameter. At this time, the P_80 variant had the lowest ORP value (339.6 mV), while the stuffing with a 1% addition of vegetable extract had the highest (P_80_M1; 358.5 mV). After 21 days of ripening, the highest ORP value was observed for the P_180 variant (367.8 mV; *p* < 0.05), and this trend was maintained until the 81st day of sausage ripening. In the last test period, the average ORP values for the variants with the addition of dandelion were significantly higher (*p* < 0.05) compared to the variant with the same dose of nitrite. A similar increase in ORP values was observed by Wójciak and Dolatowski [32] and Okoń et al. [29]. The increase in ORP values during ripening may have been due to the presence of H_2_O_2_, which may have been formed through the activities of the meat microflora and subsequently acted as a catalyst for the oxidation reaction [33]. In addition, Wójciak et al. [34] observed an analogous increase in ORP values during the storage of fermented meat products, with a stabilization of the values as the storage time increased. The addition of dandelion caused a significant (*p* < 0.05) increase in ORP values immediately after production (Day 1), while after the ripening process, the values of this parameter for these variants were significantly lower compared to the control sample (P_150). Similar test results during the use of a plant additive for fermented meat products were also obtained by Neffe-Skocińska et al. [35].

The addition of dandelion led to an increase in the water activity of the products during the initial manufacturing period (Day 1 and Day 21), particularly significant in the variant with the highest dose of dandelion extract (1%; P_80_M1). However, by Day 51, a significantly (*p* < 0.05) lower water-activity value was observed in the P_150 variant (containing a dose of 150 mg/kg of nitrite), with no significant differences between the other products. On the 81st day of ripening, no differences were observed between the sausage test variants (*p* > 0.05). The decrease in aw values observed in this study during the ripening of pork sausages aligns with trends reported in the literature by Sun et al. [36], Álvarez et al. [37], and Xiao et al. [25]. This reduction in water activity occurs due to the influence of sodium chloride and the drying process to which raw-ripening sausages are subjected [38]. The water activity value stabilized 51 days after sausage production, except for the P_150 variant, which showed a statistically significant decrease (*p* < 0.05) in water activity value. Similar results were obtained by Kononiuk and Karwowska [39]. The decrease in water activity values is beneficial for the microbiological safety of the finished product [38], inhibiting the development of undesirable microflora such as *Pseudomonas* (av < 0.97), *Clostridium botulinum* (av < 0.93–0.96), *Salmonella* (av < 0.94), and *Listeria monocytogenes* (av < 0.92). After the sausage production ripening process in all tested variants, lower aw values were obtained, which are less favorable for the development of pathogenic microorganisms [25,37,39].

### 2.3. Assessment of Protein Profile

**Figure 1:**
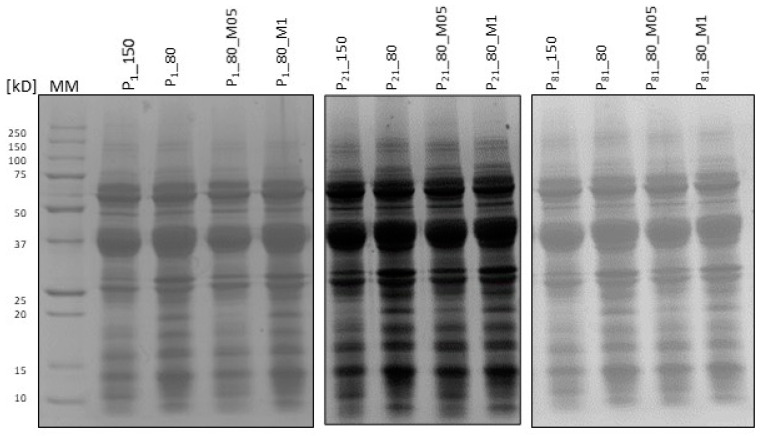
SDS-PAGE Electrophoresis result.

**Figure 2:**
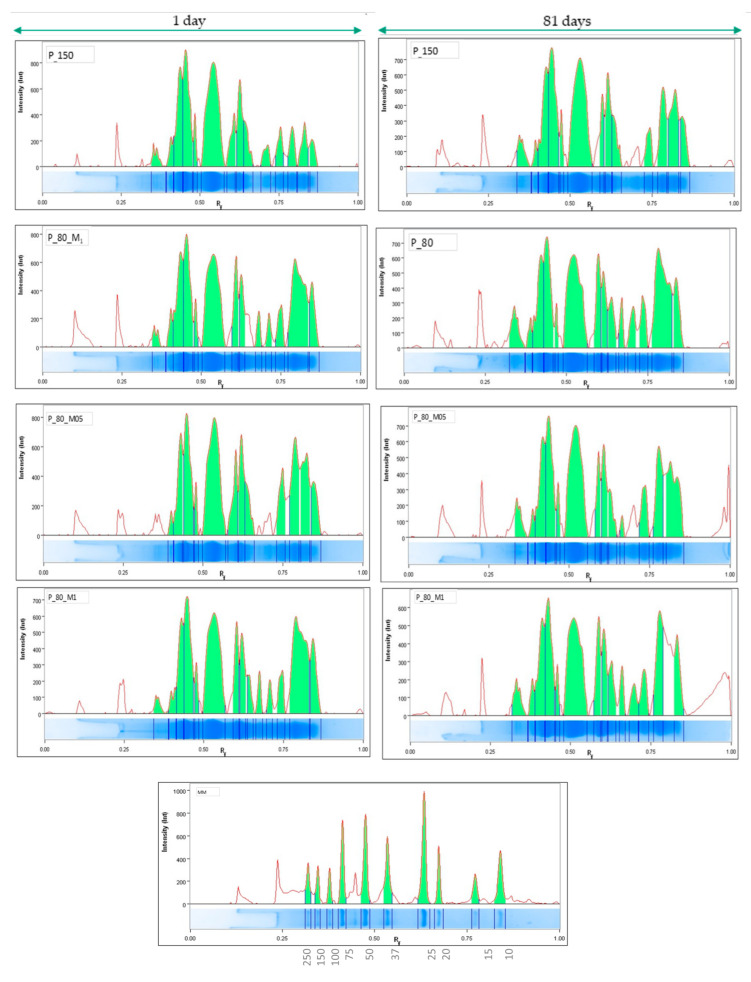
Representative densitogram of water-soluble proteins (*X*-axis means Rf; *Y*-axis means MM—molecular mass).

**Figure 3:**
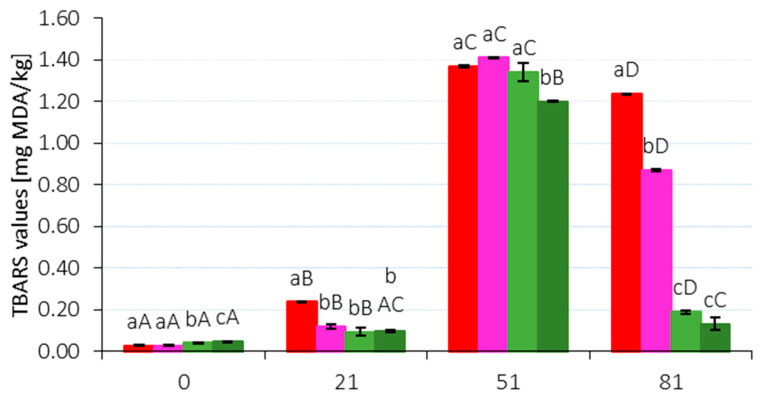
Changes in the TBARS index (mg/kg) for raw-ripening sausage during 81 days of storage (4 °C). Followed by different capital letters A–D within the same sample or by small letters a–c within different samples are significantly different (*p* < 0.05).

The process of meat ripening (1, 21, and 81 days) indicates the intensification of protein degradation processes, and the described trends correspond to the results of electrophoretic analysis, where protein extracts were divided into individual fractions (bands) according to the size criterion (250–15 kDa) (Figure 1). Figure 2 shows electrophoretograms demonstrating the separation of proteins extracted from ripening sausages immediately after production (Day 1) and after 82 days of production. During the first test period, the highest number of bands with high color intensity was found in the case of proteins extracted from the control sample (P_150) (Figure 1 and Figure 3), and the lowest in the case of variants with the addition of dandelion (taking into account the charge (vol) of the protein material for individual bands). The most intense bands of about 65–50 kDa and about 37 kDa were the most prominent on the gel path, regardless of the test variant analyzed or the ripening time. Indeed, hydrolysis of sarcoplasmic proteins occurs gradually during ripening, and the main changes observed in the 50–25 kDa range for raw ripening meats have been reported by other authors [40,41]. In general, there was no significant effect of the plant additive on the protein content in the final product, which appeared to be the same as previous studies on the effect of dandelion on the peptide profile of sous-vide-treated beef. However, after 21 and 81 days of ripening, the accumulation of protein elements with high molecular weight (about 250 kD) was observed (Figure 1 and Figure 2, and Table 4). The appearance of large protein particles in the ripening product may indicate the formation of protein aggregates rather than being the result of proteolysis. The highest level of band charge (vol; Table 4) was observed in products with reduced nitrite (P_80), suggesting an increase in protein aggregates caused by oxidation due to the use of too-low nitrite concentration. On the other hand, the intensity of staining bands of about 250 kDa (Figure 1) and the level of band charge with protein material (Table 4) characterizing meat products with dandelion are similar to those of the control variant (P_150). It suggests compensating for the reduced antioxidant effect of nitrite, caused by the use of a lower dose, by the presence of natural antioxidant compounds and antioxidants against proteins, reducing their ability to aggregate. The results of Hu et al. [42] indicate that cross-linking between proteins (actin, in that study) extracted from beef with the addition of tea polyphenols can be induced by disulfide bonding, which is consistent with the reports of Guo et al. [43]. Furthermore, it was noted that the formation of protein aggregates depended on the concentration of polyphenols [42]. In general, the use of polyphenols caused changes in protein structure due to interactions between them. The addition of polyphenols did not change the content of amino groups but caused a slight increase in the content of carbonyl groups with a simultaneous decrease in the number of sulfhydryl (SH) groups. In contrast, Cheng et al. [44] studied the effects of phenolic compounds (rutin, quercetin, and caffeic acid) on protein oxidation in Cantonese sausage during 60 days of storage at room temperature (25 ± 1 °C). The results demonstrated that the three phenolic compounds inhibited the oxidation of sarcoplasmic proteins by delaying carbonylation, the conversion of SH groups to S–S, and the formation of dimeric tyrosine and Schiff bases.

The accumulation of compounds around 17–10 kDa was also observed in the variants of the samples after ripening. These observations are the result of natural proteolysis, and the appearance of bands in the gel with increased charge (Table 4) may be due to the presence of smaller peptides. Regarding small protein elements, as shown in the representative densitogram (Figure 2), a different protein profile in the range of 17–10 kDa was observed in the variant with 1% dandelion addition (see Rf 0.75–1) with increasing product ripening time.

### 2.4. Assessment of Antiradical Potential of Raw-Ripening Pork Sausage

The solubility of phytochemical compounds, and hence their separation coefficients between aqueous and lipid phases, largely determine their localization and molecular interactions with substrates in complex food matrices [45]. Therefore, in our analysis of their effects on raw-ripening pork sausage, we chose to evaluate antioxidant activity in different extracts (alcoholic and aqueous, which also contain a fraction of sarcoplasmic proteins). Given the numerous reports highlighting the biological activity of peptides from meat products, we also included these compounds in the analysis.

Polyphenols are recognized as natural antioxidants due to their antiradical action through direct uptake. Their mechanism of action is based on their ability to transfer the hydrogen atom (·H) from hydroxyl groups to free radicals, forming a relatively stable phenolic radical. This process delays the initiation and propagation of oxidative reactions in food products. Apart from their antiradical effects, these natural phenolic compounds can also chelate the pro-oxidative transition metal cations (such as Fe^2+^ and Cu^2+^ found in muscle tissue), forming stable complexes at coordination sites between the two ring structures [46]. This ability allows polyphenols to effectively inhibit lipid and protein oxidation, thereby extending the shelf life of meat products [47].

Table 5 and Table 6 demonstrate that the primary antiradical potential was associated with the alcohol fraction, which contains a significant amount of polyphenols, compared to the water fraction. In addition, an increase in the antiradical activity of the alcohol fraction against ABTS^+^ radicals was observed over the analyzed period (Day 1 vs. Day 81; Table 4), while there was a concurrent decrease in this activity against DPPH for the alcohol fraction. Considering the water fraction, a decrease in the ability to neutralize both ABTS^+*^ and DPPH free radicals was noted over time (Table 6).

The present study demonstrated the effect of technological treatment involving fortification of the meat product with dandelion leaf extract on the antiradical potential of raw-ripening sausage. Variants with dandelion addition exhibited a higher capacity to neutralize free radicals ABTS^+*^ (except for alcoholic extracts on Day 1) as well as DPPH compared to the variant with reduced nitrite (P_80; *p* < 0.05). The results were either similar to the control variant P_150 (*p* > 0.05) or significantly higher (*p* < 0.05). Moreover, the presence of bioactive compounds of plant origin in the meat product enhanced the antiradical activity of the peptides. The biological activity of these peptides, defined as the ability to neutralize free radicals, averaged at 18.08% against ABTS^+*^ and 21.29% against DPPH after 81 days of ripening.

The statistical analysis of the TBARS index is shown in Figure 3. Immediately after production (Day 1), a significant increase in the TBARS index was observed in variants with plant additives, depending on the dose.

The P_80_M1 sample showed the highest statistically significant value (0.047 mg/kg; *p* < 0.05), while the P_150 and P_80 variants had the lowest value (0.028 mg/kg). After a ripening period of 21 days, a statistically significant (*p* < 0.05) increase in the analyzed values was observed for all samples, with the most pronounced increase seen in the control variant P_150 compared to the other samples. The increase was least pronounced in the samples with 1% dandelion addition, and this trend continued until the 51st day of maturation. Similar results were also reported by Lorenzo et al. [28] and Ge et al. [47]). According to these authors, the storage time of raw-ripened products significantly affects oxidative changes in the product, with the observed increase in TBARS attributed to the accumulation of fat decomposition products. The significant (*p* < 0.05) decrease in TBARS index values recorded in this study for all analyzed samples between 51 and 81 days of ripening was also observed by Lorenzo et al. [28], Ge et al. [48], and Okoń et al. [29]. Samples with dandelion addition had the lowest TBARS values, supporting the hypothesis of its antioxidant properties. This finding aligns with previous scientific reports [12,14].

### 2.5. Microbiological Profile

According to Weiss et al. [49], in food products such as meat, which contain high levels of protein and fat, phenols can interact with meat proteins or fat, reducing their antimicrobial activity against target bacterial cells. Therefore, the microbial profile of pork sausages ripened with dandelion was analyzed.

**Figure 4:**
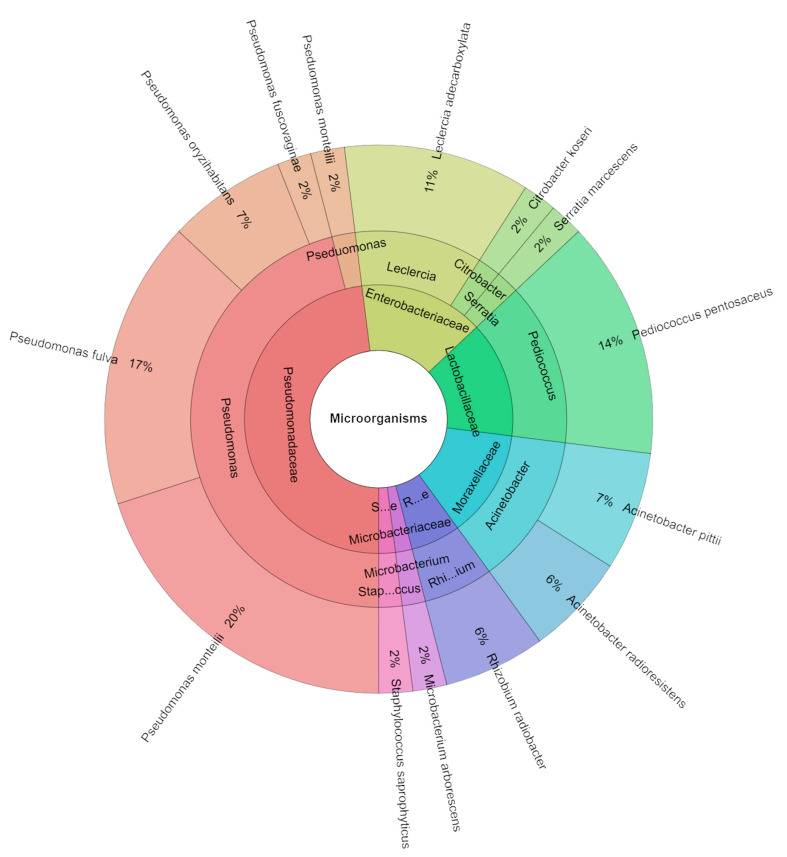
Krona chart of identified species of microorganisms from samples containing 80 mg/kg of sodium nitrite and 1% dandelion extract (P_80_M1).

The occurrence of native microorganisms with exceptional technological traits in raw materials is crucial for spontaneous fermentation [50]. These microorganisms must be capable of rapid proliferation to serve as starter cultures and counteract the growth of undesirable spoilage and pathogenic bacteria [51,52]. The total viable count in samples containing 80 mg/kg of nitrate and 1% dandelion extract ranged from 3.30 to 3.72 log CFU/g, the number of lactic acid bacteria (LAB) ranged from 3.78 to 4.04 log CFU/g, and coliform bacteria ranged from 2.95 to 3.26 log CFU/g (Table 7). A total of 212 isolates were identified in the samples containing 80 mg/kg of nitrate and 1% dandelion extracts, representing seven families, nine genera, and 14 species (Figure 4). The most commonly isolated species were *Pseudomonas monteilii* (20%), followed by *Pseudomonas fulva* (17%) and *Pediococcus pentosaceus* (14%). During the ripening period, the total aerobic population counts of the nitrate pickling salt (NP) samples were found to be lower than those of the other samples. The sausages turned redder when NP was added to the salami. It was discovered that the salamis with NP had superior microbiological quality characteristics than the other salt varieties [53]. Honikel et al. [54] found that nitrite prevents or retards microbial growth in meat products. Although the intrinsic properties of meat can influence the growth and survival of various microbial groups involved in salami fermentation, Franciosa et al. [50] reported that the production environment may also play a crucial role in determining the origin of LAB responsible for fermentation.

Plate counts revealed that the microbial communities in all productions were primarily composed of coagulase-negative staphylococci (CNS), yeasts, and lactic acid bacteria (LAB), with *Lactobacillus* and *Staphylococcus* being the most frequently isolated microorganisms. According to microbial diversity analysis, all salamis contained representatives of the Gammaproteobacteria phylum, Moraxellaceae family, *Acinetobacter*, *Pseudomonas*, *Carnobacterium*, and *Enterococcus* [55], which is consistent with the genera isolated in our work. In our study, coliform bacteria were found. All raw materials, except for the casing, contained members of the Enterobacteriaceae family, particularly *E. coli*, although their levels decreased over time. Specifically, after 30 days of ripening, *E. coli* was not detected in horse salami. *Salmonella* spp. was never detected, and *Listeria monocytogenes* was not found in casing, fat, or the meat of wild boar or horses throughout the entire testing period. However, after 45 days of ripening, *L. monocytogenes* was detected in beef and pork flesh, and its level was below the detection threshold. These pathogens are typically present during the initial stages of salami manufacturing, but their presence is typically diminished until they vanish due to the activity of fermenting groups [56]. Starting cultures are generally considered useful for controlling the presence of pathogens [57].

**Figure 5:**
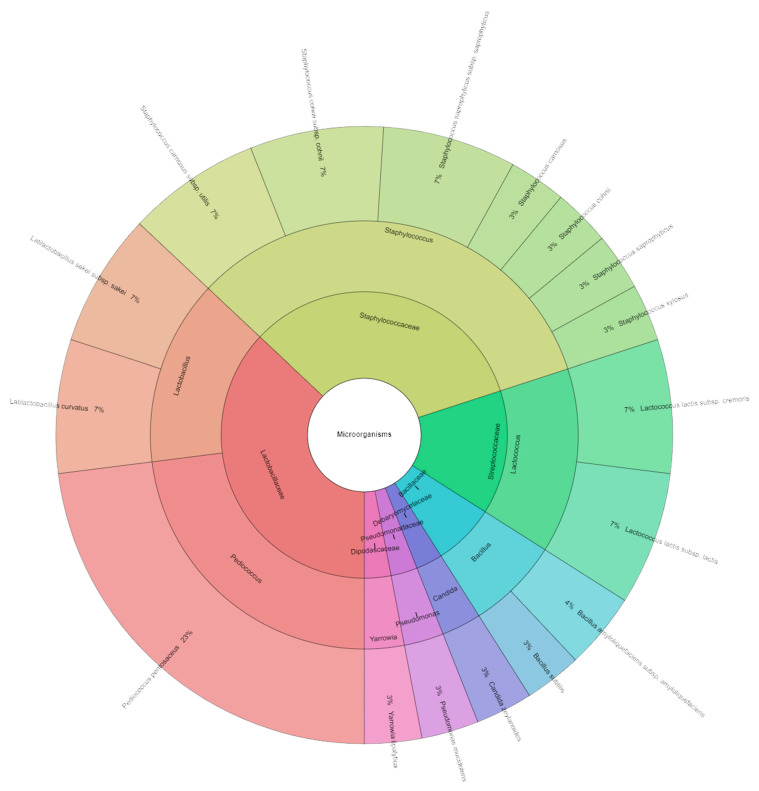
Krona chart of identified species of microorganisms from samples containing 80 mg/kg of sodium nitrite and 0.5% dandelion extract (P_80_M05).

In samples containing 80 mg/kg of nitrite and 0.5% dandelion extract, the total viable count ranged from 2.82 to 3.60 log CFU/g, the number of lactic acid bacteria ranged from 3.90 to 4.18 log CFU/g, and coliform bacteria were not found (Table 8). A total of 147 isolates were identified in samples containing 1% dandelion extract and 80 mg/kg of nitrate, representing seven families, eight genera, and 17 species. The most commonly isolated species from these samples was *Pediococcus pentosaceus* (23%) (Figure 5).

**Figure 6:**
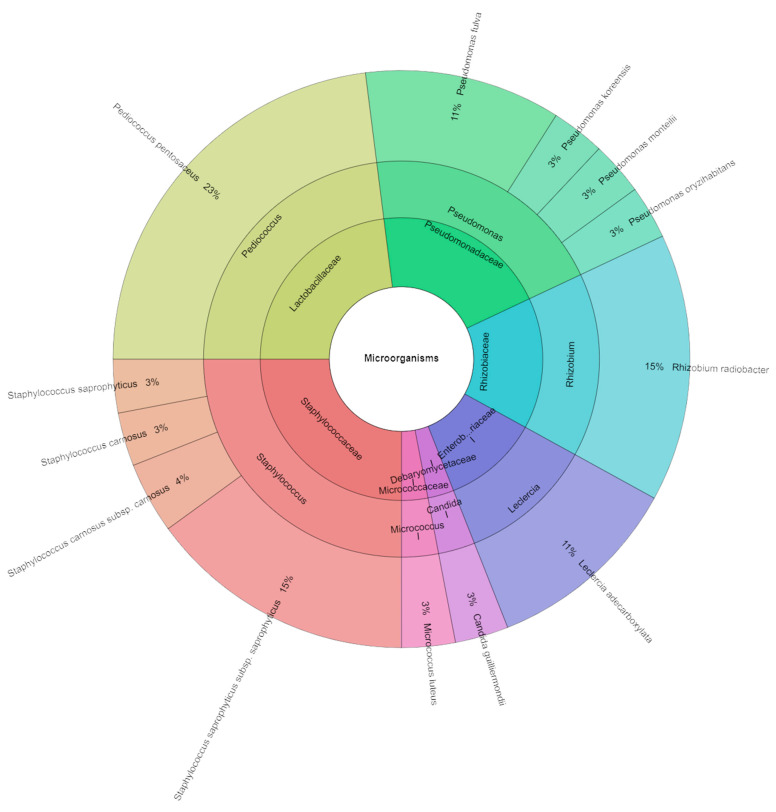
Krona chart of identified species of microorganisms from samples containing 150 mg/kg of sodium nitrite (P_150).

Samples containing 150 mg/kg of sodium nitrate III exhibited total viable counts ranging from 2.86 to 3.56 log CFU/g, 3.88 to 4.04 log CFU/g for lactic acid bacteria, and 2.90 to 3.08 log CFU/g for coliform bacteria (Table 9). In samples containing 80 mg/kg of nitrate and 1% dandelion extracts, 140 isolates were identified, representing seven genera, 13 species, and seven families. The most commonly isolated species from samples containing 150 mg/kg of sodium nitrate III was *P. pentosaceus* (23%), followed by *Rhizobium radiobacter* and *Staphylococcus saprophyticus* subsp. *saprophyticus* (14% in the samples with 150 mg/kg of sodium nitrate) (Figure 6). Lactic acid bacteria (LAB) and coagulase-negative staphylococci (CNS) are key microorganisms contributing to the stability and safety of fermented meat products [58]. According to Aquilanti et al. [59], Cocolin et al. [60], and Talon and Leroy [61], *Lactobacillus sakei*, *Lactobacillus curvatus*, and *Lactobacillus plantarum* are typically the technologically significant LAB in fermented beef products. Among CNS, *Staphylococcus xylosus* is the most common species in salamis, while Marty et al. [62] have also linked *Staphylococcus epidermidis*, *Staphylococcus equorum*, and *S*. *saprophyticus* to these products. In our study, the LAB isolates included *L*. *curvatus*, *L*. *sakei* subsp. *sakei*, *Lactococcus lactis* subsp. *cremoris*, *L. lactis* subsp. *lactis*, and the staphylococci isolates included *S. carnosus*, *S. carnosus* subsp. *utilis*, *S. cohnii*, *S. cohnii* subsp. *cohnii*, *S. saprophyticus*, *S. saprophyticus* subsp. *saprophyticus*, and *S. xylosus*. *L. sakei*, one of the most abundant species observed in the study by Settanni et al. (2020), was found to be dominant in each of the four salami species. The dominance of this species in the production of sausages [63] and several Italian salamis [59,64] is well known.

**Figure 7:**
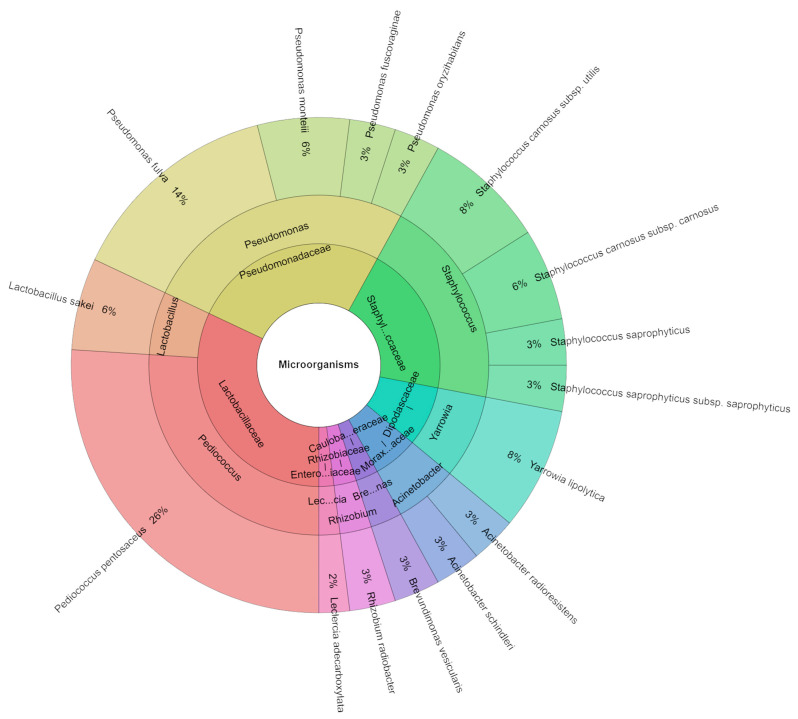
Krona chart of identified species of microorganisms from samples containing 80 mg/kg sodium nitrite.

In samples containing 80 mg/kg of nitrite, total viable counts ranged from 2.61 to 3.30 log CFU/g, lactic acid bacteria ranged from 3.70 to 4.04 log CFU/g, and coliform bacteria ranged from 2.78 to 3.30 log CFU/g (Table 10). A total of 173 isolates were identified in samples with 80 mg/kg of nitrate, representing nine families, nine genera, and 16 species. The most frequently isolated species from these samples were *P*. *pentosaceus* (26%) followed by *P*. *fulva* (14%) (Figure 7).

In the study by Pini et al. [65] on naturally dry-fermented sausages, two distinct natural extracts were employed in place of nitrites: grape seed extract (GSE) and chestnut extract (CHE). Samples with CHE demonstrated lower pH levels, which correlated with a higher relative abundance of Lactobacillaceae compared to samples with NIT and GSE. Despite substantial differences in the bacterial community and other chemical/physical characteristics among the three groups, the use of natural extracts did not significantly affect these parameters. This suggests that GSE and CHE could be used as nitrite substitutes in dry-fermented sausages. The microbiological purity of salami and other fermented foods relies heavily on the interplay of environmental factors during production [66].

## 3. Materials and Methods

### 3.1. Plant Material and Extract Preparation

The plant material used in the study consisted of dandelion (*T*. *officinale*) leaves, which were collected in April 2023 before flowering. The leaves were initially dried in a shaded, airy location and then in a dryer at 60 °C. The dried leaves were used to prepare a water extract. Ultrasound-assisted extraction (10 min) with hot distilled water (90 °C) at a plant-to-solvent ratio of 1:10 (*m*/*v*) was used. The resulting infusions were filtered after 30 min, cooled, frozen at −18 °C, and then lyophilized. Freeze drying was carried out for 72 h using a freeze dryer (Free Zone 12 lyophilizer, Labconco Corporation, Kansas City, MO, USA, 2016) at −80 °C and 0.04 mbar. The dry extract was stored in airtight plastic containers protected from light at room temperature until analysis.

### 3.2. Determination of Antioxidant Capacity of Water Extract from Dandelion Leaves

#### 3.2.1. Antioxidant Activity

The lyophilized extract was dissolved in water to achieve a starting concentration of 1 mg/mL, which was then diluted to create a series of samples with concentrations ranging from 0.1 to 1 mg/mL.

The ABTS radical cation (ABTS^·+^) [2,2-azinobis(3-ethyl-benzothiazoline-6-sulfonate)] scavenging activity was determined according to Re et al. [67]. A working solution of ABTS^·+^ radical was prepared by mixing ABTS (7 mM) with potassium persulfate (2.45 mM). The solution was kept in the dark at room temperature for 18 h and then diluted with 95% ethanol to an absorbance of 0.70 (±0.02) at 734 nm. A sample (20 μL) and ABTS^·+^ solution (3 mL) were mixed and incubated at room temperature for 10 min. The absorbance was measured at 734 nm using a Cary 50 spectrophotometer (Varian, Palo Alto, CA, USA, 2004). The control was prepared by adding 20 μL of methanol instead of the sample.

The antiradical capacity against the 2,2-diphenyl-1-picrylhydrazyl (DPPH) radical was also measured as per the method by Brand-Williams et al. [68]. Briefly, 0.1 mL of the extract solution was mixed with a freshly prepared methanolic solution of DPPH (0.1 mM, 4 mL). The mixture was vortexed and incubated in the dark at room temperature for 30 min. The absorbance was measured at 517 nm using a Cary 50 spectrophotometer (Varian, Palo Alto, CA, USA, 2004). The control was prepared by diluting 0.1 mL of methanol (analytical grade) in 4 mL of 0.1 mM DPPH.

The percentage of absorbance reduction regarding the initial value was used to calculate the inhibition percentage, according to the formula %AA = [1 − (Ap/Ab)] × 100%, where AA is the antioxidant activity of the analyzed sample, Ap is the absorbance of the analyzed sample, and Ab is the absorbance of the blank sample. The EC_50_ values were evaluated based on the dependence between antiradical activity and sample concentration *f*(c) = %AA.

#### 3.2.2. Determination of Total Phenolic Content (TPC)

The total phenolic content of the extracts was analyzed using the Folin–Ciocalteu method with gallic acid (25–500 mg/L) as a standard, following the procedure outlined by Singleton and Rossi [69]. The extracts (0.06 mL) were mixed with distilled water (0.54 mL), 1.5 mL of diluted FC’s reagent (1:10 with distilled water), and 1.2 mL of sodium bicarbonate solution (7.5%). The samples were then incubated in darkness at room temperature for 30 min before measuring absorbance at 750 nm using a Cary 50 spectrophotometer (Varian, Palo Alto, CA, USA, 2004). The results were expressed as mg gallic acid equivalent (GAE) per gram of dry extract.

#### 3.2.3. Determination of Total Flavonoids

Total flavonoids were determined using a spectrophotometric method based on the formation of a colored complex between the flavonoid and AlCl_3_ [70]. Lyophilized extract dissolved in water at a starting concentration of 1 mg/mL was used. The analyzed samples (0.25 mL), ethanol (0.75 mL—96%), AlCl_3_ (0.05 mL—10%), sodium acetate (0.05 mL—1 M), and distilled water (1.4 mL) were mixed for the determination. Absorbance was measured at a wavelength of 415 nm. Total flavonoids were expressed as quercetin equivalents based on a calibration curve prepared previously for this compound. The results were presented as the means of four replications in mg of quercetin/g dry extract.

#### 3.2.4. Extract Preparation for Vitamin C Content Determination

Vitamin C was determined using the spectrofluorimetric method described by Xia Wu [71] with some modifications. The following reagents were used: 3% metaphosphoric acid, 7 M HCl, 0.1 M Na_2_S_2_O_3_, and 0.005 M H_2_SO_4_. The oxidizing solution was prepared by dissolving 1.3 g of I2 in 10 mL of 40% KI, adding 0.1 mL of 7 M HCl, and diluting with distilled water to a final volume of 100 mL. A 0.1 M Na_2_S_2_O_3_ solution was prepared by dissolving 1.25 g of the reagent and 0.01 g of Na_2_CO_3_ in 50 mL of water. The derivatization reagent was prepared by dissolving 10 mg of o-phenylenediamine (OPDA) in 10 mL of 0.005 M H_2_SO_4_.

Lyophilized extracts of dandelion leaves (0.2 g) were diluted in 10 mL of 3% metaphosphoric acid. For the analysis, 2 mL of sample extract or L-ascorbic acid standard (50–500 μg/L) was mixed with 0.3 mL portions of a 0.005 M iodine solution in potassium iodide, vortexed for 1 min, and then supplemented with 0.3 mL portions of 0.01 M Na_2_S_2_O_3_. The pH of the samples was adjusted to approximately 6.0 by adding 0.3 mL of 2 M NaOH, and the derivatization was carried out by adding 0.3 mL portions of the OPDA solution. The solutions were stirred for 30 min at maximum stirred force. Afterward, the solutions were diluted to 50 mL with distilled deionized water. The determinations were performed using a Cary Eclipse spectrofluorimeter (Varian, Palo Alto, CA, USA, 2004) at an excitation wavelength of λ = 365 nm and an emission wavelength of λ = 425 nm. The vitamin C content was calculated based on a standard curve obtained using an aqueous solution of L-ascorbic acid standard. The results were expressed as μg L-ascorbic acid per 100 g of sample (dry basis).

#### 3.2.5. LC-MS Analysis

The LC-QTOF-MS method was utilized for detailed investigations of constituents in water extracts from dandelion leaves. The analysis was carried out using a liquid chromatograph (Agilent Technologies 1290, 2020) with an MS detector (Agilent 6530 Accurate-Mass Quadrupole Time-of-Flight, 2020). A Zorbax C-18 column (1.8 μm, 2.1 mm × 50 mm), maintained at 40 °C, was used (Agilent Technologies, Palo Alto, CA, USA). The mobile phase consisted of 1% acetic acid in acetonitrile (A) and water (B) in a gradient proportion of solvents, where the concentration of solvent A was varied from 20% to 90% over 19 min. The flow rate was set at 0.4 mL/min, and the sample injection volume was 5 μL. Mass spectra were acquired in the mass range of 100–2000 Da in both positive (ESI+) and negative (ESI−) ionization modes. Data were processed using MassHunter software (https://www.agilent.com.cn/en/promotions/masshunter-mass-spec, accessed on 29 April 2024) (Agilent Technologies, Palo Alto, CA, USA).

## 4. Meat Product Analysis

### 4.1. Preparation of the Meat Product

The study focused on raw-ripening pork sausages, which were produced using a mixture of pork ham (80%) and pork jowl (20%). The meat and fat were treated and cured using specially prepared curing mixtures with reduced nitrite content. The meat stuffing was prepared by grinding the meat ingredients and mixing them with the additives included in the product recipe (Table 11). Moguntia-brand starter cultures called BessaSTART were used in the production of raw-ripening sausage. The mixture declared by the manufacturer included *S. xylosus*, *S. carnosus*, and *P. pentosaceus*. These cultures were added at a rate of 30 g/50 kg of raw meat. Dandelion extract (*T. officinale*) was included in two variants, with P_80_M05 containing 0.5% and P_80_M1 containing 1% dandelion. Glucose (0.6%) was added during mixing as a medium for the starter cultures. The meat stuffing was filled into fibrous casings to create sausages weighing between 0.4 and 0.5 kg. The sausages were then placed in a ripening chamber where the ripening process continued for 3 weeks. During this process, parameters such as temperature and relative humidity were controlled. After 21 days, the sausages were vacuum-packed and stored at a refrigeration temperature of 4 °C.

### 4.2. Physicochemical Parameters

#### 4.2.1. Total Acidity

The parameter was tested according to PN-ISO 2917:2001 [72]. A 10 g crushed sample was homogenized with 100 mL of distilled water for 60 s. To measure acidity, a CPC-505 pH/digital conductivity meter (Elmetron, Zabrze, Poland, 2009) equipped with an ERH-111-type combined pH electrode was used. The electrode’s readings were established, and the result obtained was read with an accuracy of 0.01 units of pH value.

#### 4.2.2. Oxidation-Reduction Potential

This index was tested according to the method proposed by Nam and Ahn [73]. A 10 g crushed sample was homogenized with 100 mL of distilled water for 60 s. A CPC-505 pH/digital conductivity meter (Elmetron, Zabrze, Poland, 2009) equipped with an ERPt-11-type platinum compound electrode was used for the measurement. The result obtained from the measurement was converted into the value of the ORP relative to the standard hydrogen method *E*_h_ [mV].

#### 4.2.3. Water Activity

Water activity (a_w_) was measured using a Novasina LabMaster (Novasina AG, Lachen, Switzerland 2015) following the manufacturer’s instructions at 25 °C. A 5 g test sample was placed in a measuring vessel and closed with a lid. The lid was removed immediately before placing the test sample in the apparatus. After the measurement was completed, the result was read from the instrument’s display to the nearest 0.001.

### 4.3. TBARS Level

The TBARS index was determined to study the oxidative stability of ripening sausages by measuring the reactivity of thiobarbituric acid [74]. For the determination, 10 g of crushed raw material was used, to which 50 mL of 7.5% cold trichloroacetic acid (TCA) was added, after which the mixture was homogenized using an IKA T25 homogenizer (IKA-Werke GmbH & Co., KG, Staufen, Germany). The homogenate was then shaken for half an hour and drained. Subsequently, 5 mL of the filtrate was collected and mixed with an equal volume of 2-thiobarbituric acid, followed by heating for 40 min in a water bath heated to 100 °C. After the heating process, the samples were cooled, and 5 mL of chloroform was added. The TBARS content were measured using U-5100 UV-VIS spectrophotometer (HITACHI High America Inc., Schaumburg, IL, USA, 2014). The blank consisted of a solution of 5 mL of TBA, 5 mL of TCA, and 5 mL of chloroform.

### 4.4. Electrophoretic Separation

The protein samples were separated on SDS-PAGE gels according to the Laemmli method [75]. The electrophoretic separation conditions were as follows: 5% stacking gel and 14% separating gel at a constant current of 50 V for the stacking gel and 100 V for the separating gel. A mass marker in the range of 250–15 kDa (Bio-Rad, Hercules, CA, USA, 2012) was used for the comparative analysis of bands. Five microliters of the marker were loaded into the first lane, and 20 μg of protein was loaded into subsequent lanes. Protein content was evaluated in aqueous extracts of sausages using the Bradford method. The analysis was performed using the Mini Protean Tetra Cell (Bio-Rad, Hercules, CA, USA). After the decolorization process, protein degradation products were identified by their molecular weights, estimated from their relative electrophoretic mobility compared to molecular weight standards. The amounts of each peptide band were calculated relative to the optical density using the Gel DocTM EZ densitometer scanner imaging system and Image Lab software ver. 4.1 (Bio-Rad, Hercules, CA, USA). The intensity of each band was calculated as its actual intensity relative to the intensity of the marker band (mass marker).

### 4.5. Evaluation of Antiradical Activity

#### 4.5.1. Obtaining the Extracts and Peptides

In the experiment, alcoholic and aqueous extracts were obtained from the meat product. A 5 g sample was crushed and homogenized with 20 mL of either ethanol or distilled water using an IKA T25 homogenizer from Staufen, Germany. The resulting homogenate was then centrifuged at 5000× *g* for 30 min at 4 °C and filtered using filter paper. The extracts prepared in this manner were stored at −20 °C for further analysis.

Peptide preparation was carried out according to the method of Mora et al. [76] In this process, 5 g of the sample was homogenized with 20 mL of 0.01 M HCl for 1 min while cooling on ice using a homogenizer. The homogenate was then centrifuged at 5000× *g* for 30 min at 4 °C. After filtration, 5 mL of the supernatant was added to 15 mL of frozen ethanol. The mixture was kept at 4 °C overnight and then centrifuged at 5000× *g* for 30 min at 4 °C. The resulting supernatant was collected and concentrated in an evaporator by evaporating the alcohol. The concentrated extract was dissolved in 0.01 M HCl, filtered through a 0.45 μm nylon membrane filter (AlfaChem, Torun, Poland), and stored at −20 °C before use.

#### 4.5.2. Antiradical Activity

The antiradical properties of the obtained extracts (alcoholic, aqueous, and peptide) were measured using an ABTS^+•^ cation radical and DPPH radical test, following the method described by Jung et al. [77]. The absorbance at 734 nm (ABTS) or 517 nm (DPPH) was measured using a UV-visible spectrophotometer (Nicolet Evolution 300, Thermo Electron Corp., Waltham, MA, USA). The scavenging ability was determined using the following formula:

Radical-scavenging activity (%) = (1 − *A*_2_/*A*_1_) × 100,

where *A*_1_ is the absorbance of the control sample, and *A*_2_ is the absorbance of the sample.

### 4.6. Microbiological Analysis

Samples of 5 g each were mixed with 45 mL of a 0.1% sterile saline solution in an Erlenmeyer flask. The blending process took place for 30 min in a shaking incubator (GFL 3031, Burgwedel, Germany). The evaluation included the examination of various microorganisms. Coliforms were identified in the Violet Red Bile Lactose Agar coliform bacterial culture medium (VRBL, Oxoid, Basingstoke, UK) when incubated at 37 °C for 24–48 h. Total viable counts (TVC) were determined using Plate Count Agar (PCA; Oxoid, Basingstoke, UK) and cultured at 30 °C for 48–72 h. Lactic acid bacteria were cultured on De Man Rogosa and Sharpe Agar (MRS; Oxoid, Basingstoke, UK) for 48–72 h at 37 °C. Each analysis and test was conducted in triplicate. Additionally, a brief reinoculation at 30 °C involved culturing eight colonies per Petri dish on Tryptone Soya agar (TSA; Oxoid, Basingstoke, UK).

#### 4.6.1. Identification of Microorganisms Using Mass Spectrometry

The identification of microorganisms isolated from beef meat samples was carried out using the MALDI-TOF MS Biotyper (Bruker, Daltonics, Bremen, Germany) along with reference libraries.

#### 4.6.2. Preparing the MALDI-TOF Matrix Solution

The MALDI-TOF Matrix Solution originated as a stock solution and later transformed into an organic substance. The standard solution comprised 50% acetonitrile, 47.5% water, and 2.5% trifluoroacetic acid. To create 1 mL of the stock solution, 500 mL of pure 100% acetonitrile, 475 mL of filtered water, and 25 mL of pure 10% trifluoroacetic acid were blended. The “HCCA matrix portioned” was prepared in a 250 mL Eppendorf flask and mixed with the organic solvent. All matrix components were procured from Aloqence in Vrable, Slovakia.

#### 4.6.3. Identification of Microorganisms

The sample preparation adhered to previous recommendations, which involved selecting eight distinct colonies on the Petri plate. The biological material was then transferred from the Petri plate to an Eppendorf flask containing 300 mL of distilled water, followed by the addition of 900 mL of ethanol and thorough mixing. The resulting mixture underwent centrifugation using a ROTOFIX 32A (Ites, Vranov, Slovakia) at 10,000× *g* for 2 min. After removing the supernatant, the precipitate was extracted from the Eppendorf tube and air-dried at room temperature (20 °C). Next, 30 mL of 70% formic acid and 30 μL of acetonitrile were added to the particles. After centrifugation at 10,000× *g* for 2 min, 1 μL of the liquid was applied to a MALDI plate, followed by the addition of 1 μL of MALDI matrix solution. The samples were dried, and a MALDI-TOF mass spectrometer (Bruker, Daltonics, Bremen, Germany) was used for microorganism detection. Mass spectra were automatically generated by the microflex LT MALDI-TOF mass spectrometer (Bruker Daltonics, Bremen, Germany) in the linear positive mode, spanning a mass range of 2000–20,000 Da. Calibration of the devices was performed using the Bruker bacterial test standard. The acquired mass spectra were evaluated using the MALDI Biotyper 3.0 program (Bruker Daltonics, Bremen, Germany). The identification criteria were as follows: a score below 1.700 was considered unreliable for identification, a score between 2.300 and 3.000 indicated highly probable species identification, a score between 2.000 and 2.299 suggested genus identification with plausible species identification, and a score between 1.700 and 1.999 indicated probable genus identification.

### 4.7. Statistical Analysis

All analyses were carried out in four replicates, and standard deviations were calculated for each data series to indicate the scatter plots of the dataset. The significance of the differences between the averaged values of the different variants was determined from the one-way analysis of variance at the level of significance α ≤ 0.05. Differences in averages were verified using the Tukey test. Statistical comparisons were carried out using STATGRAPHIC Centurion software, version XVI (Statgraphics Technologies, Inc., The Plains, VA, USA).

## 5. Conclusions

Nitrite is a multifunctional additive that is commonly used in the meat industry. However, due to the negative effect of nitrite on human health, it is important to reduce the amount of nitrite added to meat products. The obtained research results indicate that dandelion leaf extract (*Taraxacum officinale*) is rich in phenolic acids, flavonoids, coumarins and their derivatives (LC-QTOF-MS method); at 0.5% and 1%, it improved the oxidative stability of raw-ripening sausages, reducing the nitrite content to 80 mg/kg. A higher quantity of extract increases the antioxidant property of the product. The addition of dandelion to raw-ripening pork sausages did not significantly affect the microbiological profile of the products. The hygiene of the production process has the biggest impact on the microbiological quality of the products. Moreover, the addition of dandelion extract to meat products, in combination with a lower quantity of nitrite, did not cause any negative effects on the products’ quality (pH value, water activity, or protein profile). This study demonstrates that it is possible to use low quantities of nitrite in the preparation of meat products, and that the addition of dandelion extract helps to achieve a product quality similar to that achieved using sodium nitrite alone. The major difficulty lies in choosing the optimal quantity of plant extract to apply. Therefore, further studies are still needed in this regard.

## Figures and Tables

**Table 1:** Chemical content and antioxidant activity of water extract from dandelion leaves.

Analyzed Parameters	
Extraction yield (%)	30.61 ± 0.11
Vitamin C (mg/100 g)	5.39 ± 0.01
DPPH (EC50, μg/mL)	68.4 ± 0.15
ABTS (EC50, μg/mL)	36.5 ± 0.05
Total phenolic content (mg gallic acid/g)	303.2 ± 4.7
Flavonoids content (mg quercetin/g)	18.06 ± 0.14

*n* = 3 ± SD.

**Table 2:** Profile of phenolic compounds in dandelion-leaf water extract determined with the LCMS-ESI-QTOF method positive (ESI+) ionization modes.

No	*R*_t_	Name *	Molecular Formula	M_obs_	M_teor_	*m*/*z*	Diff (ppm)
**1**	1.274	Neochlorogenic acid	C_16_H_18_O_9_	354.0958	354.0951	355.1028	1.93
**2**	1.307	Caffeoyltartaric acid	C_13_H_12_O_9_	312.0484	312.0481	313.0556	0.79
**3**	1.307	7-hydroxycoumarin	C_9_H_6_O_3_	162.0317	162.0317	163.039	0.23
**4**	1.623	Cichoriin	C_15_H_16_O_9_	340.08	340.0794	341.0874	1.6
**5**	2.772	Esculetin	C_9_H_6_O_4_	178.0274	178.0267	179.0347	4.47
**6**	3.288	Dihydrosyringin	C_17_H2_6_O_9_	374.1586	374.1577	375.1690	2.34
**7**	3.738	Taraxafolin	C_11_H_14_O_5_	226.0858	226.0841	227.093	7.39
**8**	3.821	Quercetin-3-*O*-Ara-Glc	C_26_H_28_O_16_	596.1384	596.1377	597.1455	1.19
**9**	3.855	Quercetin-3,4′-di-Glc	C_27_H_30_O_17_	626.1522	626.1563	627.1578	6.21
**10**	3.888	Quercetin-3-(malonyl-Glc)-Glc	C_30_H_32_O_20_	712.1501	712.1487	713.1567	2.03
**11**	4.021	Caffeoyl malic acid	C_13_H_12_O_8_	296.0551	296.0532	297.0623	6.27
**12**	4.337	Scopoletin	C_10_H_8_O_4_	192.0427	192.0423	193.0499	2.26
**13**	4.371	Luteolin 3′,7-*O*-di-Glc	C_27_H_30_O_16_	610.1534	610.1523	611.1635	1.22
**14**	4.504	Quercetin-3-*O*-Ara	C_20_H_18_O_11_	434.0871	434.0849	457.0769	5.15
**15**	4.504	Chlorogenic acid	C_16_H_18_O_9_	354.0954	354.0951	355.1026	0.78
**16**	4.504	L-chicoric acid	C_22_H_18_O_12_	474.0806	474.0798	475.0878	1.59
**17**	4.851	Luteolin 7-*O*-Rhamnoside	C_27_H_30_O_15_	594.1585	594.1585	595.1652	−0.87
**18**	4.903	3,5-di-*O*-caffeoylquinic acid	C_25_H_24_O_12_	516.1282	516.1023	517.1352	2.12
**19**	4.947	Luteolin 7-*O*-Glc	C_21_H_20_O_11_	448.1012	448.1006	449.1085	1.51
**20**	5.268	Rosmarinic acid	C_18_H_16_O_8_	360.084	360.0845	3383.0742	−1.33
**21**	6.073	Luteolin	C_15_H_10_O_6_	286.0456	286.0477	287.0527	−7.45

* Ara: arabinoside; Glc: glucoside.

**Table 3:** Comparison of physicochemical parameters of raw sausages during ripening.

Parameter	Time [Day]	P_150	P_80	P_80_M05	P_80_M1
pH	1	6.52 ± 0.02 ^Aab^	6.50 ± 0.01 ^Aa^	6.49 ± 0.03 ^Aa^	6.55 ± 0.01 ^Ab^
	21	5.43 ± 0.05 ^Ba^	5.44 ± 0.04 ^BCa^	5.31 ± 0.01 ^Bb^	5.26 ± 0.01 ^Bc^
	51	5.44 ± 0.02 ^Ba^	5.42 ± 0.01 ^Ba^	5.32 ± 0.03 ^Bb^	5.28 ± 0.01 ^Cc^
	81	5.46 ± 0.03 ^Ba^	5.47 ± 0.03 ^Ca^	5.38 ± 0.02 ^Cb^	5.34 ± 0.01 ^Db^
ORP [mV]	1	345.60 ± 1.21 ^Aa^	339.60 ± 1.00 ^Ab^	349.33 ± 1.49 ^Ac^	358.50 ± 2.82 ^Ad^
	21	367.8 ± 4.42 ^Ba^	353.58 ± 13.65 ^Abb^	355.62 ± 6.99 ^Aab^	361.84 ± 5.58 ^ABab^
	51	385.97 ± 10.52 ^Ca^	367.75 ± 11.03 ^BCb^	365.17 ± 6.01 ^Bb^	366.65 ± 7.30 ^Bb^
	81	387.90 ± 1.53 ^Ca^	369.65 ± 7.55 ^Cb^	377.45 ± 3.77 ^Cc^	380.90 ± 1.20 ^Cc^
aw	1	0.966 ± 0.003 ^Aa^	0.965 ± 0.002 ^Aa^	0.968 ± 0.001 ^Aab^	0.969 ± 0.001 ^Ab^
	21	0.877 ± 0.007 ^Ba^	0.878 ± 0.008 ^Ba^	0.878 ± 0.002 ^Bab^	0.889 ± 0.007 ^Bb^
	51	0.871 ± 0.011 ^Ca^	0.883 ± 0.002 ^Bb^	0.879 ± 0.009 ^Bb^	0.884 ± 0.008 ^BCb^
	81	0.863 ± 0.003 B^Ca^	0.867 ± 0.006 ^Ca^	0.873 ± 0.006 ^Ba^	0.875 ± 0.010 ^Ca^

P_150: control sausage containing 150 mg/kg of sodium nitrate; P_80: control sausage containing 80 mg/kg of sodium nitrate; P_80_M05: sausage containing 80 mg/kg of sodium nitrate and 0.5% dandelion addition; P_80_M1: sausage containing 80 mg/kg of sodium nitrate and 1% dandelion addition. With different capital letters are significantly different (*p* < 0.05) in the same column. With different small letters are significantly different (*p* < 0.05) in the same row. Values are presented as mean ± standard error (SE).

**Table 4:** Protein distribution and volume (Int) of bands in the gel after electrophoresis.

Lp.	P_0__150		P_0__80		P_0__80_M05		P_0__80_M1		P_90__150		P_90__80		P_90__80_M05		P_90__80_M1	
	MW kDa	Vol *10^3^	MW kDa	Vol *10^3^	MW kDa	Vol *10^3^	MW kDa	Vol *10^3^	MW kDa	Vol *10^3^	MW kDa	Vol *10^3^	MW kDa	Vol *10^3^	MW kDa	Vol *10^3^
1	-	-	-	-	-	-	-	-	147.5	240	170.4	356	174.8	258	203.8	249
2	131.0	*168*	135.5	*136*	-	*-*	137.8	*115*	-	*-*	-	*-*	-	*-*	-	*-*
3	76.6	*169*	80.8	*172*	83.4	*89*	79.1	*145*	87.1	*106*	90.9	*123*	93.8	*106*	96.9	*169*
4	63.9	*816*	65.0	*815*	67.2	*627*	67.2	*560*	68.9	*693*	70.7	*712*	71.9	*724*	75.0	*617*
5	57.7	*1048*	58.2	*911*	59.7	*970*	60.2	*869*	61.8	*958*	63.3	*866*	65.0	*885*	67.2	*758*
6	48.0	*170*	48.7	*149*	49.3	*143*	50.0	*149*	50.9	*167*	52.6	*124*	53.5	*155*	55.8	*125*
7	**36.1**	*1867*	37.0	*1635*	37.0	*1769*	37.8	*1528*	38.0	*1683*	39.8	*1621*	40.1	*1679*	42.0	*1418*
8	27.9	*362*	27.6	*478*	28.3	*466*	28.3	*394*	28.7	*272*	28.9	*502*	26.6	*342*	30.1	*418*
9	25.9	*572*	25.9	*390*	26.5	*557*	26.5	*397*	27	*518*	27.2	*394*	27.9	*469*	28.3	*371*
10	23.7	*330*	19.6	*155*	24.6	*338*	24.1	*168*	25.1	*323*	24.6	*337*	25.7	*256*	25.8	*291*
11	-	-	-	-	-	-	19.9	*160*	-	*-*	20.5	*200*	21.1	*66*	21.5	*175*
12	17.9	*186*	17.8	*158*	-	*-*	18.1	*138*	-	*-*	18.2	*257*	-	*-*	18.6	*189*
13	15.8	*275*	16.0	*288*	16.1	*459*	16.3	*245*	16.4	*244*	16.7	*319*	16.8	*357*	17.0	*274*
14	13.2	*294*	12.7	*1490*	13.6	*814*	13.1	*1467*	14.0	*575*	13.8	*1532*	14.3	*661*	14.4	*684*
15	10.6	*340*	-	*-*	11.0	*754*	-	*-*	11.2	*682*	-	*-*	11.3	*981*	10.5	*454*
16	10.0	*195*	10.0	*442*	10.0	*417*	10.0	*483*	10.0	*328*	10.2	*470*	-	*-*	-	*-*

**Table 5:** Antiradical effect against ABTS [%].

Parameter	Time [Day]	P_150	P_80	P_80_M05	P_80_M1
ABTS_A-E_	1	33.33 ± 4.03 ^Aa^	39.90 ± 2.56 ^Ab^	35.24 ± 0.10 ^Aa^	37.24 ± 1.23 ^Aab^
	81	45.55 ± 2.64 ^Ba^	47.79 ± 2.84 ^Ba^	52.36 ± 2.41 ^Bb^	59.98 ± 1.14 ^Bc^
ABTS_W-E_	1	41.85 ± 1.49 ^Aa^	33.54 ± 2.92 ^Ab^	41.92 ± 3.81 ^Aa^	43.54 ± 2.22 ^Aa^
	81	16.76 ± 1.03 ^Ba^	17.88 ± 0.62 ^Ba^	22.44 ± 0.72 ^Bb^	23.29 ± 1.03 ^Bb^
ABTS_PEP_	1	18.52 ± 1.20 ^Aa^	10.44 ± 1.09 ^Ab^	23.65 ± 1.80 ^Ac^	28.17 ± 1.60 ^Ad^
	81	17.85 ± 3.12 ^Aa^	10.55 1.16 ^Ab^	15.07 ± 2.78 ^Ba^	21.90 ± 2.75 ^Bc^

With different capital letters are significantly different (*p* < 0.05) in the same column. With different small letters are significantly different (*p* < 0.05) in the same row. Values are presented as mean ± standard error (SE).

**Table 7:** Microbiological quality of samples containing 80 mg/kg of nitrite and 1% dandelion extract (P_80_M1).

Sample	Total Viable Count	Lactic Acid Bacteria	Coliforms Bacteria
1	3.58	3.85	2.95
2	3.60	3.81	3.00
3	3.45	4.00	3.26
4	3.72	4.00	3.08
5	3.68	4.04	3.30
6	3.68	3.85	3.30
7	3.60	3.78	3.30
8	3.51	3.90	3.15
9	3.56	3.95	3.20
10	3.30	3.90	2.95

**Table 8:** Microbiological quality of samples containing 80 mg/kg of nitrite and 0.5% dandelion extract (P_80_M-5).

Sample	Total Viable Count	Lactic Acid Bacteria	Coliforms Bacteria
1	2.82	4.00	ND
2	3.15	4.04	ND
3	3.20	4.18	ND
4	3.26	4.04	ND
5	3.30	4.00	ND
6	3.20	4.00	ND
7	3.00	3.90	ND
8	3.20	3.95	ND
9	3.18	3.95	ND
10	3.60	4.04	ND

**Table 9:** Microbiological quality of samples containing of 150 mg/kg of nitrite (P_150).

Sample	Total Viable Count	Lactic Acid Bacteria	Coliforms Bacteria
1	2.95	3.85	ND
2	3.30	3.90	ND
3	3.38	4.00	ND
4	3.08	3.95	ND
5	2.90	4.04	ND
6	2.86	3.68	3.00
7	2.98	3.90	2.78
8	2.88	3.93	3.08
9	3.11	4.04	2.90
10	3.56	3.88	3.00

**Table 10:** Microbiological quality of samples containing of 80 mg/kg of sodium nitrite (P_80).

Sample	Total Viable Count	Lactic Acid Bacteria	Coliforms Bacteria
1	3.30	4.00	ND
2	3.26	3.85	ND
3	3.30	4.04	ND
4	2.85	4.04	ND
5	3.28	3.95	ND
6	3.26	3.70	2.78
7	3.15	3.90	2.90
8	2.61	3.95	2.85
9	3.08	3.78	3.30
10	3.04	3.85	3.18

**Table 6:** Antiradical effect against DPPH [%].

Parameter	Time [Day]	P_150	P_80	P_80_M05	P_80_M1
DPPH_A-E_	1	58.48 ± 3.77 ^Aa^	60.04 ± 0.92 ^Aa^	74.07 ± 1.66 ^Ab^	81.01 ± 0.82 ^Ac^
	81	55.59 ± 1.38 ^Ba^	56.36 ± 1.96 ^Ba^	70.01 ± 2.31 ^Bb^	84.44 ± 1.57 ^Bc^
DPPH_W-E_	1	14.83 ± 1.75 ^Aa^	13.66 0.41 ^Aa^	17.66 ± 0.60 ^Ab^	25.39 ± 0.53 ^Ac^
	81	11.71 ± 0.21 ^Ba^	13.90 ± 0.20 ^Ab^	16.63 ± 1.62 ^Ac^	18.83 ± 0.20 ^Bc^
DPPH_PEP_	1	15.92 ± 1.97 ^Aa^	16.72 ± 2.73 ^Aa^	22.58 ± 1.03 ^Ab^	24.53 ± 0.65 ^Ac^
	81	12.28 ± 1.92 ^Ba^	15.76 ± 1.54 ^Ab^	18.32 ± 1.52 ^Bb^	24.25 ± 1.71 ^Bc^

With different capital letters are significantly different (*p* < 0.05) in the same column. With different small letters are significantly different (*p* < 0.05) in the same row. Values are presented as mean ± standard error (SE).

**Table 11:** Research variants of raw-ripened sausages.

	Nitrite (mg/kg)	Dandelion (%)
P_150	150	-
P_80	80	-
P_80_M05	80	0.5
P_80_M1	80	1

## Data Availability

Data are contained within the article.

## Supplementary Materials

The following supporting information can be downloaded at: https://www.mdpi.com/article/10.3390/molecules29102249/s1, Table S1: Identification of phenolic compounds in dandelion leaf water extract determined with the LCMS-ESI-QTOF method in positive (ESI+) ionization modes. Refs. [78,79,80,81] are cited in Supplementary Materials.
