# Supplementary material for: Effect of the Addition of Dandelion (Taraxacum officinale) on the Protein Profile, Antiradical Activity, and Microbiological Status of Raw-Ripening Pork Sausage
Source: Molecules. 2024 May 10;29(10):2249. doi: 10.3390/molecules29102249 (PMC11124098; doi:10.3390/molecules29102249)
Supplement: Supplementary file 1 [file molecules-29-02249-s001.zip › molecules-2952972-supplementary.pdf]

*molecules*

MDPI

*Article*

# Effect of the Addition of Dandelion (*Taraxacum officinale*) on the Protein Profile, Antiradical Activity, and Microbiological Status of Raw-Ripening Pork Sausage

**Karolina Wójciak [1,*], Małgorzata Materska [2], Arkadiusz Pełka [1], Agata Michalska [1], Teresa Małecka-Massalska [3], Miroslava Kačániová [4,5], Natália Čmiková [4], and Mirosław Słowiński [6]**

[1] Department of Animal Food Technology, Faculty of Food Science and Biotechnology, University of Life Sciences in Lublin, Skromna 8, 20-704 Lublin, Poland

[2] Department of Chemistry, Faculty of Food Science and Biotechnology, University of Life Sciences in Lublin, Akademicka 15, 20-950 Lublin, Poland

[3] Chair and Department of Human Physiology, Medical University of Lublin, Radziwiłłowska 11, 20-080 Lublin, Poland

[4] Institute of Horticulture, Faculty of Horticulture and Landscape Engineering, Slovak University of Agriculture, Tr. A. Hlinku 2, 94976 Nitra, Slovakia; miroslava.kacaniova@gmail.com (M.K.), n.cmikova@gmail.com (N.Č)

[5] School of Medical & Health Sciences, University of Economics and Human Sciences in Warsaw, Okopowa 59, 01 043 Warszawa, Poland; m.kacaniova@vizja.pl (M.K.)

[6] Division of Meat Technology, Department of Food Technology and Food Evaluation, Institute of Food Sciences, Warsaw University of Life Sciences-SGGW, 02-787 Warsaw, Poland

* Correspondence: karolina.wojciak@up.lublin.pl; Tel.: +81 4623340

**Citation:** Wójciak, K.; Materska, M.; Pełka, A.; Michalska, A.; Małecka-Massalska, T.; Kačániová, M.; Čmiková, N.; Słowiński, M. Effect of the addition of dandelion (*Taraxacum officinale*) on the protein profile, antiradical activity, and microbiological status of raw-ripening pork sausage. *Molecules* **2024**, *29*, 2249. https://doi.org/10.3390/molecules29102249

Academic Editor: Adele Papetti

Received: 22 March 2024
Revised: 29 April 2024
Accepted: 29 April 2024
Published: 10 May 2024

**Copyright:** © 2024 by the authors. Submitted for possible open access publication under the terms and conditions of the Creative Commons Attribution (CC BY) license (https://creativecommons.org/licenses/by/4.0/).

### S.1. Qualitative analysis of dandelion extract

LC-QTOF-MS analyses were performed to estimate the qualitative composition of the tested extract. The presence of five compounds was revealed by the analysis of mass spectra of their standards. For these compounds, the LC-MS analyses were performed in the *scan* mode to confirm their retention times and in the *target* mode to confirm their fragmentation spectra. Other compounds were identified on the basis of literature data and available databases (PubChem, ChemSpider). In Table S1 precursor ions for each identified compound as well as compound CID number described in the Pub Chem database are inserted. All compounds were earlier identified in dandelion, which was confirmed by appropriate references.

**Table S1.** Identification of phenolic compounds in dandelion leaf water extract determined with the LCMS-ESI-QTOF method in positive (ESI+) ionization modes.

| No | Name* | $R_t$ | Molecular formula | Precursor type | Precursor m/z | Precursor type | Precursor m/z | PubChem Compound CID | Identification /confirmation |
|---|---|---|---|---|---|---|---|---|---|
| **1** | Neochlorogenic acid | 1.274 | $C_{16}H_{18}O_9$ | $[M+H]^+$ | 355.102 | - | - | 5280633 | [2, 3] |
| **2** | Caffeoyltartaric acid | 1.307 | $C_{13}H_{12}O_9$ | $[M+H]^+$ | 313.0556 | $[M+Na]^+$ | 335.0377 | 6440397 | [1, 2] |
| **3** | 7-hydroxycoumarin | 1.307 | $C_9H_6O_3$ | $[M+H]^+$ | 163.039 | - | - | 5281426 | [4] |
| **4** | Cichoriin | 1.623 | $C_{15}H_{16}O_9$ | $[M+H]^+$ | 341.0871 | $[M+Na]^+$ | 363.0589 | 442101 | [4] |
| **5** | Esculetin | 2.772 | $C_9H_6O_4$ | $[M+H]^+$ | 179.0347 | - | - | 5281416 | [4] |
| **6** | Dihydrosyringin | 3.288 | $C_{17}H2_6O_9$ | $[M+H]^+$ | 375.169 | $[M+Na]^+$ | 397.147 | 71720642 | [4] |
| **7** | Taraxafolin | 3.738 | $C_{11}H_{14}O_5$ | $[M+H]^+$ | 227.093 | - | - | 10987942 | [4] |
| **8** | Quercetin-3-*O*-Ara-Glc | 3.821 | $C_{26}H_{28}O_{16}$ | $[M+H]^+$ | 597.1455 | - | - | 5484066 | [1] |
| **9** | Quercetin-3, 4′-di-Glc | 3.855 | $C_{27}H_{30}O_{17}$ | $[M+H]^+$ | 627.1578 | $[M+Na]^+$ | 649.1403 | 5320835 | [1] |
| **10** | Quercetin-3-(malonyl-Glc)-Glc | 3.888 | $C_{30}H_{32}O_{20}$ | $[M+H]^+$ | 713.1576 | $[M+Na]^+$ | 735.1489 | 74978238 | [1] |
| **11** | Caffeoyl malic acid | 4.021 | $C_{13}H_{12}O_8$ | $[M+H]^+$ | 297.0623 | $[M+Na]^+$ | 319.0429 | 6124299 | [3] |
| **12** | Scopoletin | 4.337 | $C_{10}H_8O_4$ | $[M+H]^+$ | 193.0499 | $[M+Na]^+$ | 215.0306 | 5280460 | [4] |
| **13** | Luteolin 3′, 7-*O*-di-Glc | 4.371 | $C_{27}H_{30}O_{16}$ | $[M+H]^+$ | 611.1635 | $[M+Na]^+$ | 633.1416 | 5490298 | [1] |
| **14** | Quercetin-3-*O*-Ara | 4.504 | $C_{20}H_{18}O_{11}$ | $[M+H]^+$ | 435.0931 | $[M+Na]^+$ | 457.0769 | 12309865 | [1, 2] |
| **15** | Chlorogenic acid | 4.504 | $C_{16}H_{18}O_9$ | $[M+H]^+$ | 355.1026 | $[M+Na]^+$ | 377.0855 | 1794427 | Standard, [1, 3] |
| **16** | L-chicoric acid | 4.504 | $C_{22}H_{18}O_{12}$ | $[M+H]^+$ | 475.0878 | $[M+Na]^+$ | 497.0702 | 5281764 | [1, 2, 3] |
| **17** | Luteolin 7-*O*-Rhamnoside | 4.851 | $C_{27}H_{30}O_{15}$ | $[M+H]^+$ | 595.1652 | $[M+Na]^+$ | 617.1558 | 4636593 | Standard, [1, 2] |
| **18** | 3,5-di-*O*-caffeoylquinic acid | 4.903 | $C_{25}H_{24}O_{12}$ | $[M+H]^+$ | 517.1352 | - | - | 6474310 | [1] |
| **19** | Luteolin 7-*O*-Glc | 4.947 | $C_{21}H_{20}O_{11}$ | $[M+H]^+$ | 449.1085 | $[M+Na]^+$ | 471.0867 | 5282149 | Standard,[1,2,3] |
| **20** | Rosmarinic acid | 5.268 | $C_{18}H_{16}O_8$ | - | - | $[M+Na]^+$ | 383.0742 | 5281792 | [4] |
| **21** | Luteolin | 6.073 | $C_{15}H_{10}O_6$ | $[M+H]^+$ | 587.0527 | - | - | 5280445 | Standard, [3] |

Glc: glucoside, Gal: galactoside, Ara: arabinoside

## S2. Q-TOF spectra for standard compounds

### Rosmarinic acid

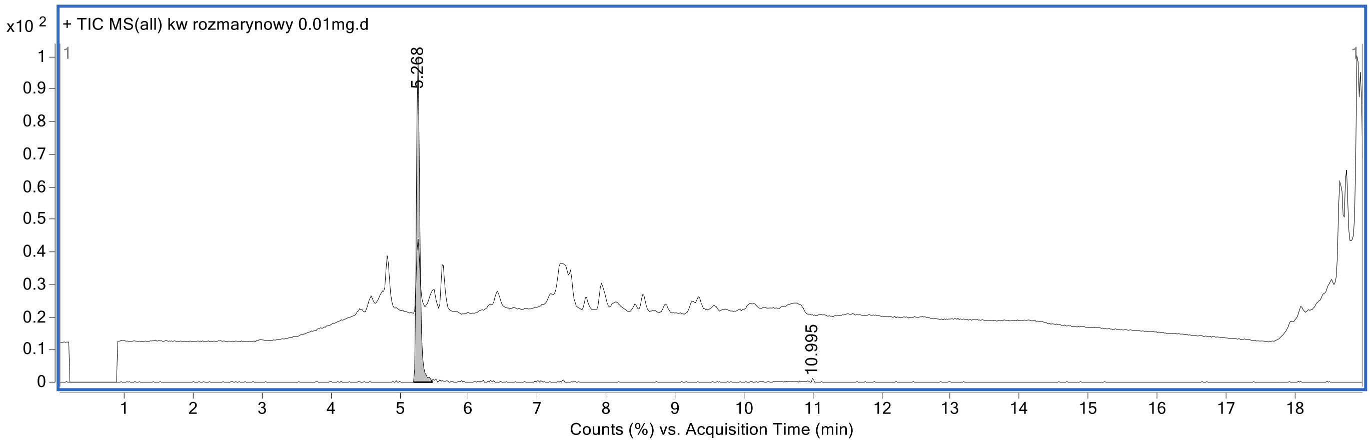


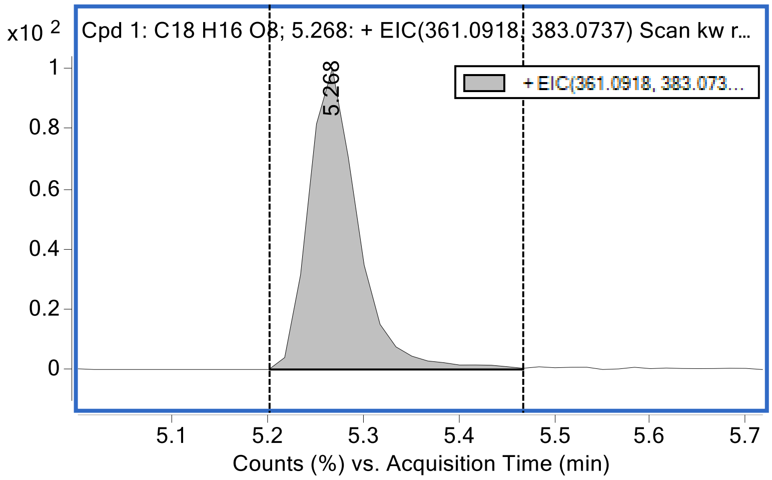


### Chlorogenic acid

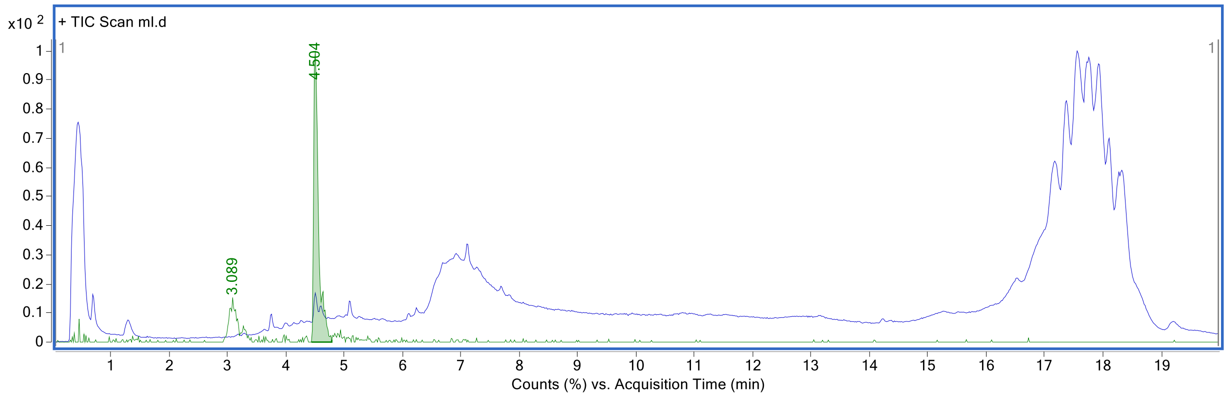

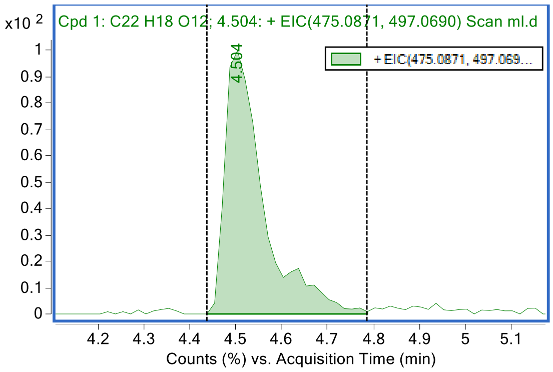


**Luteolin 7-O-rhamnoside**

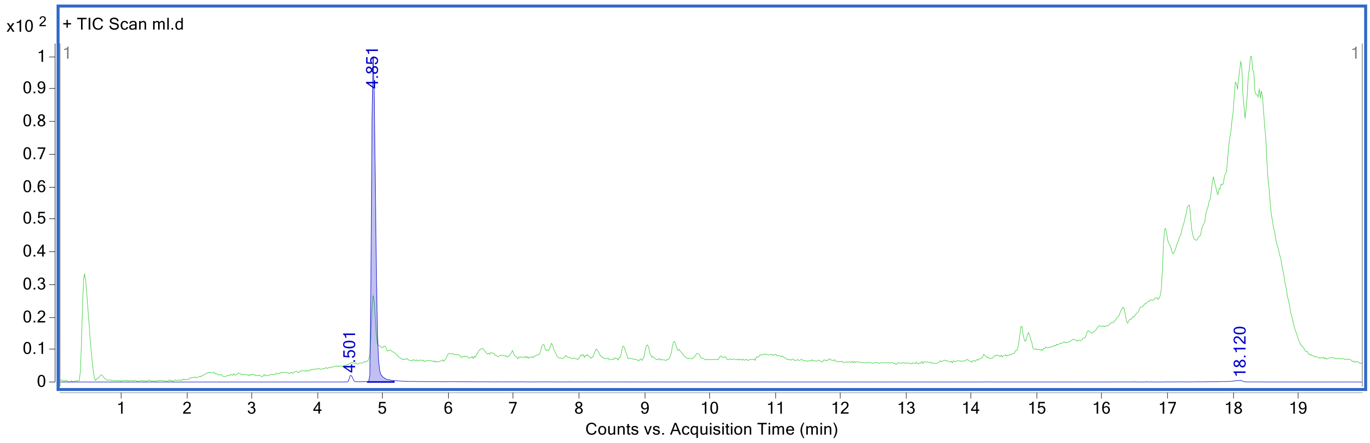


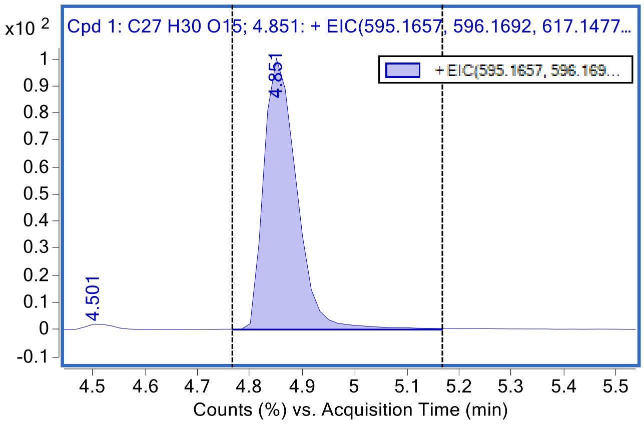

**Luteolin 7-O-glucoside**

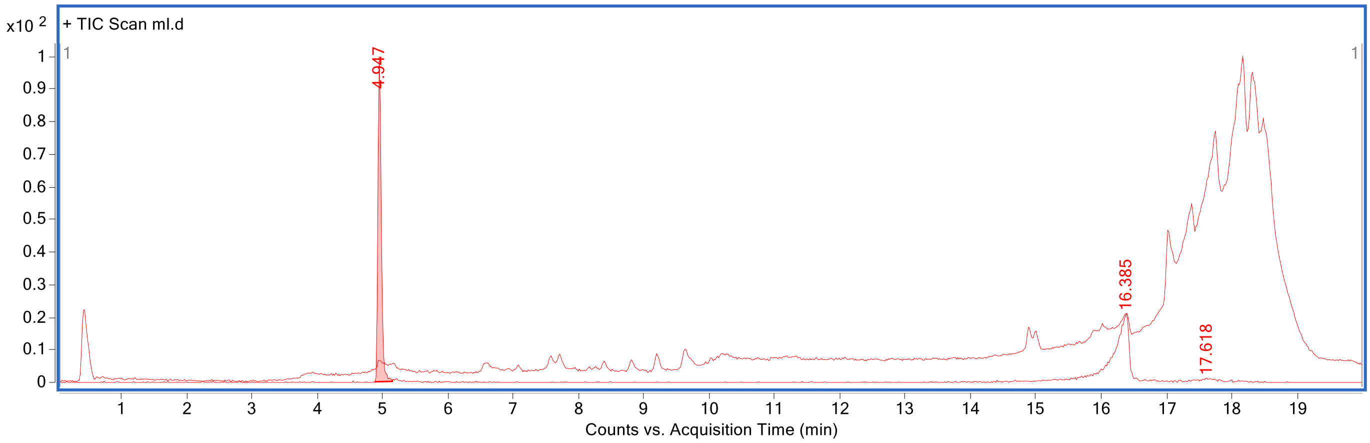


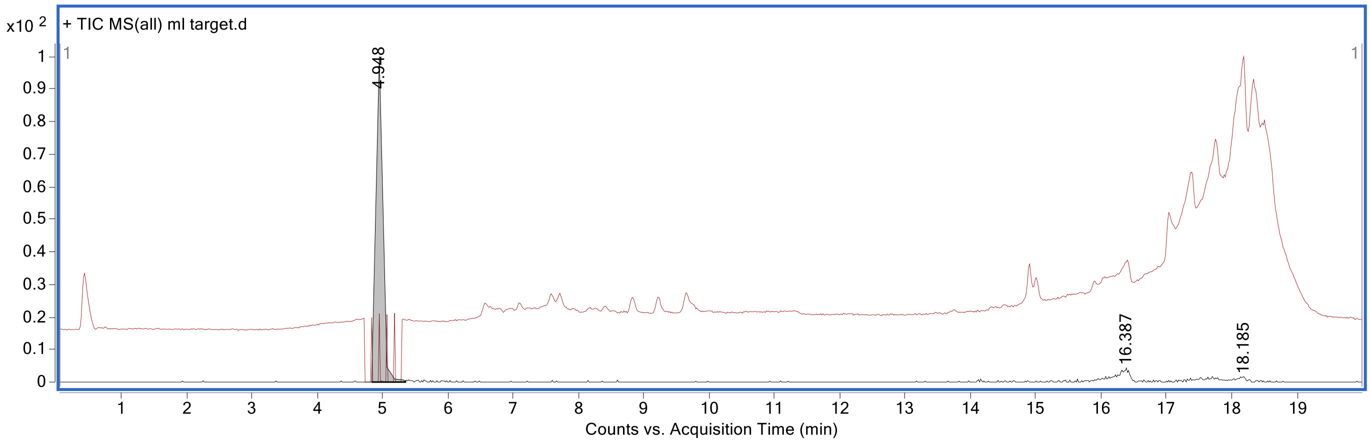


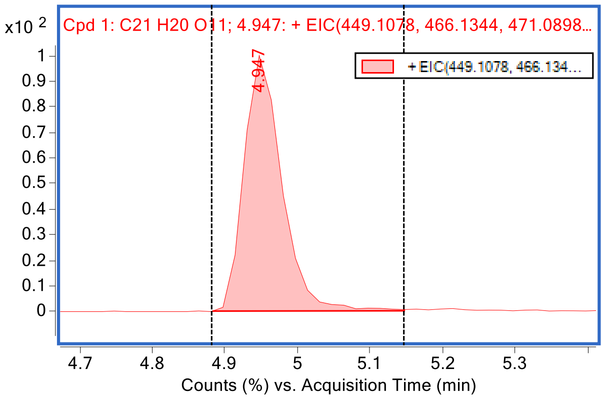

**Luteolin**

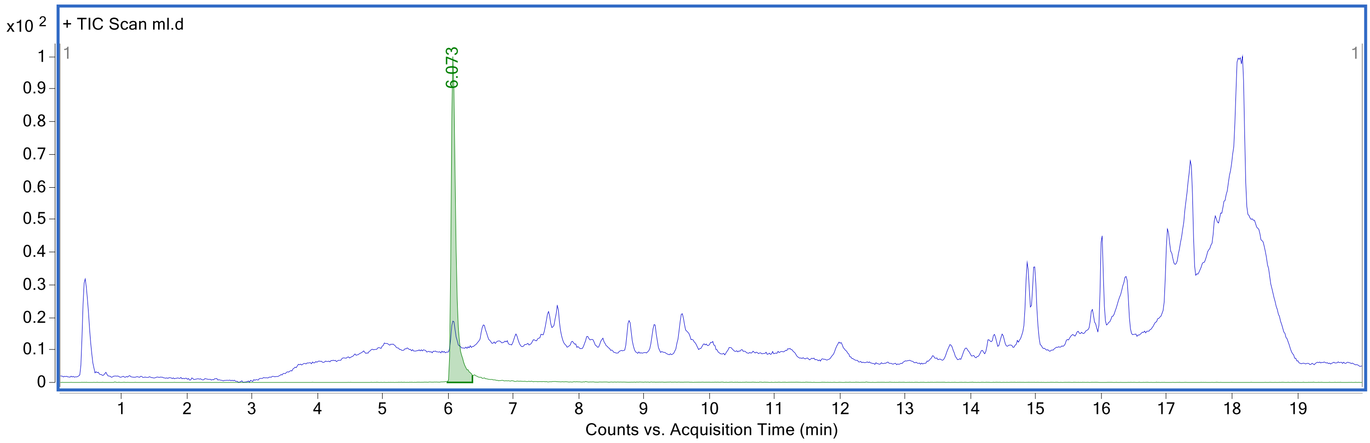
x10 2
+ TIC Scan ml.d
6.073
Counts vs. Acquisition Time (min)

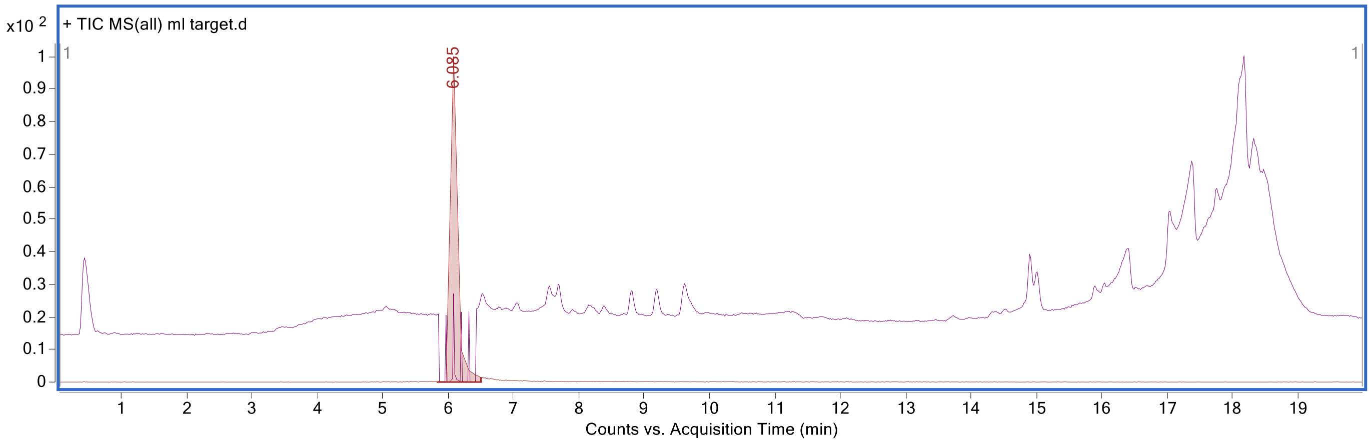
x10 2
+ TIC MS(all) ml target.d
6.085
Counts vs. Acquisition Time (min)

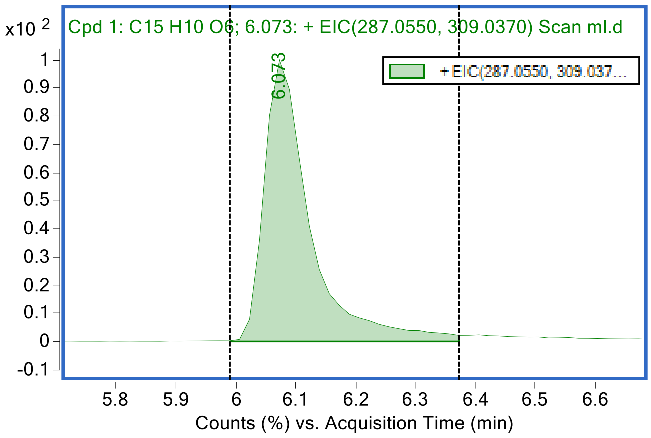
x10 2
Cpd 1: C15 H10 O6; 6.073: + EIC(287.0550, 309.0370) Scan ml.d
6.073
+EIC(287.0550, 309.037...
Counts (%) vs. Acquisition Time (min)

**References**

[1]. Chen H.J, Inbaraj B.S., Chen B.H. Determination of phenolic acids and flavonoids in *Taraxacum formmosanum* Kitam by liquid chromatography- tandem mass spectrometry coupled with a post-column derivatization technique. *Int. J. Mol. Sci.* 2012, *13*, 260-285; doi: 10.3390/ijms13010260.

[2]. Diaz M.I., Barros L., Alves R.C., Oliveira M.B., Santos-Buelga C., Ferreira I., Nutritional composition, antioxidant activity and phenolic compounds of wild Taraxacum sect. Ruderalia. *Food Res. Int*. 2014, 56, 266-271. Doi: 10.1016/j.foodres.2014.01.003

[3]. Miłek M., Marcincakova D., Legath J. Polyphenols content, antioxidant activity and cytotoxic assessment of Taraxacum officinale extracts prepared through the micelle-mediated extraction method. *Molecules*, 2019, 24, 1025, DOI: 10.3390/molecules24061025

[4]. Fan M., Zhang X., Song H., Zhang Y. Dandelion (Taraxacum genus): A review of chemical constituents and pharmacological effects. Molecules, 2023, 28, 5022, doi: 10.3390/molecules28135022
